# Models of animal coalitions and their implications for human evolution

**DOI:** 10.1098/rspb.2024.1227

**Published:** 2024-10-30

**Authors:** Yasuo Ihara

**Affiliations:** ^1^ Department of Biological Sciences, The University of Tokyo, Hongo 7-3-1, Bunkyoku, Tokyo 113-0033, Japan

**Keywords:** three-player coalition game, social selection, *Ardipithecus ramidus*, primate behaviour, revolutionary coalition, conflict intervention

## Abstract

Social interaction is a prime driver for the evolution of animal behaviour. Dyadic interaction, in particular, has been the focus of intensive research on the evolution of mutualistic, altruistic, selfish or spiteful behaviours. Meanwhile, triadic interaction has been the minimal framework for the study of animal coalition as observed in some species of primates, as well as in carnivores and cetaceans, where two or more individuals act jointly against a third party in a competitive context. Previous mathematical models of animal coalition have either failed to explain the observed diversity in the configuration of coalition or presumed fine-tuned decision-making that may be unrealistic for non-human animals. To approach these issues, the present study develops a new model that is fairly simple, but still able to account for the observed diversity in animal coalitions. Analysis of the model specifies key parameters affecting the predicted types of coalition: the nature of the benefit being contested, the cost-to-benefit ratio associated with fighting and the synergistic effect in coalition formation. Additionally, the model is used to evaluate the social selection hypothesis, which claims that coalition formation induced social selection favouring reduced aggression and lower fighting abilities during human evolution.

## 1. Introduction

Social interactions between individuals have played a major role in the evolution of animal behaviour. As the most fundamental building block, interactions between two individuals have been the focus of numerous investigations, both theoretical and empirical, on the evolution of animal behaviour. One common way to classify social behaviour in dyadic contexts, which goes back to Hamilton [1], is based on its effect on the fitness of the interacting individuals. A behaviour is mutualistic if it improves both the actor’s and the recipient’s fitness, and selfish if it enhances the actor’s fitness at the cost of the recipient’s fitness. A behaviour that is costly to the actor is altruistic if it is beneficial to the recipient, and spiteful if it is also costly to the recipient [2].

A new dimension that opens up when we extend our view beyond dyadic interactions is coalition formation. Animal coalition typically refers to two or more individuals acting jointly against a third party in an aggressive or competitive context [3]. It is a form of cooperation in competition and most commonly manifested as an individual’s intervention in an ongoing conflict to support one party or the other [4]. In female philopatric species, coalitions formed by females may be understood as a nepotistic support based on maternal kinship, producing stable matrilineal hierarchies, whereas the role of relatedness tends to be limited in the formation of male–male coalitions [5]. Since a coalition makes sense only in the presence of three or more individuals, triadic social interaction has been the minimal framework for the study of coalition formation [3,6–8]. Animal coalition is worth studying partly because it may have placed adaptive demands on some species, perhaps including humans, thereby promoting the evolution of triadic awareness [9–11] and the capacity of sharing intentions with others to establish the ‘we-mode’ [12].

Animal coalitions are often classified, according to the relative dominance of interacting individuals, into three types [5,13]. A coalition formed by dominant individuals against a lower-ranking target is called a conservative, or all-down, coalition. A bridging coalition is formed by individuals ranking above and below a target. In a revolutionary, or all-up, coalition, subordinate individuals jointly attack a higher-ranking target.

In a socioecological context in which revolutionary coalition is a feasible option, it likely hinders dominant individuals from monopolizing vital resources, or may even exclude them from the circle of cooperation. This may bring about social selection [14,15] that favours physically weaker or psychologically less aggressive individuals (hereafter, the social selection hypothesis [16–18])

A possible example of social selection that acted in the human lineage is found in *Ardipithecus ramidus*, whose upper canine teeth were reduced in size and shape, with the CP3 complex, the honing structure to continuously sharpen the upper canines, being lost [19,20]. Since canine teeth function as a weapon in within-group conflicts in male primates [21,22], the dentition of *Ardipithecus* is thought to suggest a reduction in male aggression, probably associated with a fundamental shift in social behaviour [19,23]. Another finding that may be indicative of selection for reduced aggression and enhanced social tolerance in the human lineage is the feminization of craniofacial morphologies in *Homo sapiens* since the Middle Pleistocene [24]. The latter observation is often attributed to the process of human self-domestication, which is claimed to have occurred uniquely in *H. sapiens* [25–27]; for example, Wrangham [27] argued that coalitionary killing of bullies, which may have substantiated selection against aggression [17], became possible only in *H. sapiens* owing to their ability of language-based conspiracy.

Pandit & van Schaik [28] and van Schaik *et al.* [13] developed one of the most influential models to study coalition formation in male primates. In their framework, joining a coalition may enhance a male’s reproductive success either by mitigating the mating skew in favour of high-ranking males (i.e. levelling coalitions [28]) or by helping him rise in the dominance hierarchy (i.e. rank-changing coalitions [13]). A coalition is predicted to form if it is both profitable and feasible, where a coalition is profitable if all the participants gain a net benefit, and feasible if it is strong enough to defeat the top-ranking male, only in which case the coalition is assumed to be able to modify the allocations of mating opportunities. The model provides testable predictions about the occurrence of levelling and rank-changing coalitions of various sizes and configurations. A limitation of the Pandit–van Schaik model is that it precludes by assumption any conservative coalitions from being profitable. However, as exemplified in table 1, male primates do form conservative coalitions, actually more frequently than revolutionary coalitions, even though a considerable variation exists both between and within species. For attempts to extend the original Pandit–van Schaik model to account for the occurrence of conservative coalitions, see Pandit *et al.* [43] and Toyoda *et al.* [44].

**Table 1.** Observed numbers and proportions of conservative, bridging and revolutionary coalitions in male primates.

species	conservative		bridging		revolutionary		total	source
*Chlorocebus pygerythrus*	15	(0.24)	19	(0.31)	28	(0.45)	62	Freeman *et al.* [29]
*Macaca assamensis*	70	(0.71)	24	(0.24)	5	(0.05)	99	Young *et al.* [30]
*Macaca fuscata*	20	(0.57)	11	(0.31)	4	(0.11)	35	Kawazoe [31]
*Macaca fuscata*	16	(0.80)	0	(0)	4	(0.20)	20	Kutsukake & Hasegawa [32]
*Macaca mulatta*	6	(0.86)	0	(0)	1	(0.14)	7	Higham & Maestripieri [33]
*Macaca mulatta*	11	(0.41)	1	(0.04)	15	(0.56)	27	Higham & Maestripieri [33]
*Macaca mulatta*	29	(0.83)	5	(0.14)	1	(0.03)	35	Kulik *et al.* [34]
*Macaca nigra*	88	(0.69)	24	(0.19)	16	(0.13)	128	Neumann *et al.* [35]
*Macaca radiata*	594	(0.50)	435	(0.37)	159	(0.13)	1188	Silk [36]
*Macaca sylvanus*	8	(0.09)	29	(0.32)	52	(0.58)	90	Bissonnette *et al.* [37]
*Macaca sylvanus*	29	(0.18)	62	(0.40)	64	(0.41)	155	Berghänel *et al.* [38]
*Macaca sylvanus*	68	(0.41)	73	(0.44)	24	(0.15)	165	Widdig *et al.* [39]
*Macaca sylvanus*	19	(0.54)	7	(0.20)	9	(0.26)	35	Young *et al.* [30]
*Macaca sylvanus*	45	(0.55)	27	(0.33)	10	(0.12)	82	Young *et al.* [30]
*Macaca sylvanus*	63	(0.36)	98	(0.55)	15	(0.08)	177	Young *et al.* [30]
*Macaca thibetana*	21	(0.78)	5	(0.19)	1	(0.04)	27	Berman *et al.* [40]
*Macaca thibetana*	32	(0.84)	2	(0.05)	4	(0.11)	38	Berman *et al.* [40]
*Pan troglodytes^a^*	1	(0.08)	4	(0.31)	8	(0.62)	13	Boesch & Boesch-Achermann [41]
*Pan troglodytes^b^*	6	(0.32)	8	(0.42)	5	(0.26)	19	Nishida & Hosaka [42]

^a^ Seventeen cases of male–female coalitions are excluded.

^b^ Four cases with ambiguous male ranks are excluded.

Another group of models consider, as a minimal framework to study animal coalitions, a competition over resource among three individuals [18,45–51]. Here, we briefly review three of them. First, Mesterton-Gibbons & Sherratt [47] analysed a game-theoretic model of coalition formation among three individuals. In their model, each individual has a randomly assigned strength value, which affects the propensity to win a competition over a resource. Each individual is also characterized by a threshold value as a heritable strategy, with which the individual’s own strength is compared. Knowing only its own strength, an individual attempts to form a coalition if its strength is below the threshold; otherwise, it chooses to fight alone. When only one or no individual attempts to form a coalition, the three individuals fight independently for the resource. When two and only two individuals attempt to form a coalition, they fight jointly against the third individual, and if they win, the resource is equally divided between them. When all individuals attempt to form a coalition, there is no fighting and a three-way equal division of the resource follows. Assuming that both coalition attempt and fighting impose some costs on individuals, the model identifies three different types of evolutionarily stable states, in which it is predicted that every individual always fights alone, all individuals always share the resource without fighting or only the two weakest individuals may form a coalition, depending on parameter values. Thus, the model never predicts conservative or bridging coalitions.

Second, a more recent work by Stamatopoulos *et al.* [49] formulated the triadic interaction as an extensive-form game, in which an individual decides whether and whom to solicit for coalition formation, and in case a solicitation is made it is either accepted or rejected by the individual being solicited. The procedure is repeated until a coalition is successfully formed or all possible coalitions are rejected. The probability to win a fight increases with the relative strength, where a coalition’s strength is assumed to be equal to the sum of the strengths of the coalition members. If an individual wins a fight, the resource is obtained by that individual, while if a coalition wins, the resource is allocated in proportion to the strengths of the coalition members. Under the assumption that individuals make decisions to maximize their payoffs based on the knowledge of the strengths of all individuals, it was shown that the game has a unique subgame perfect equilibrium, in which conservative, bridging or revolutionary coalition is predicted, depending on the relative strengths of the individuals. Thus, Stamatopoulos *et al*.’s model successfully explains the range of observed diversity in animal coalitions. In doing so, however, the model assumes a considerably complex extensive-form game, requiring sophisticated decision-making by animal individuals, the plausibility of which seems debatable.

Third, Ihara [18] explored the social selection hypothesis using another model of the three-player coalition game. As in the two earlier studies mentioned above, the game is about competition over resource among three individuals with different resource-holding potentials (RHPs). A conflict breaks out when an individual decides which of the two other individuals to attack, after which the third individual chooses from three options: to help the attacker, to help the one being attacked or to remain outside the conflict. Whenever the third individual decides to provide support, a coalition is formed against the lone individual, and if the coalition wins a battle, the resource is divided between the coalition members in proportion to their RHPs. When no help is provided, on the contrary, the first two individuals fight against each other, and whoever wins will fight a second battle with the third individual to determine the final winner. Analysis of subgame perfect equilibria revealed that the model predicts revolutionary coalitions, but not conservative or bridging coalitions. It was also found that the expected payoff of the highest-ranking (as measured by the RHP) individual can be lower than those of the intermediate- and/or lowest-ranking individuals under certain conditions, indicating that social selection is a viable hypothesis in animal species whose socioecology can be approximated by this model.

These and other studies of the three-player coalition game have demonstrated that simple models can explain basic mechanisms underlying observed diversity in animal coalitions, make predictions to be tested empirically and evaluate the plausibility of novel hypotheses. On the other hand, as the above brief review of the literature has already revealed, some predictions from coalition games seem to depend critically on details of the model and may be altered in a non-trivial way by subtle changes in the assumptions. For example, Stamatopoulos *et al.* [49] and Ihara [18] both considered an extensive-form coalition game involving three heterogeneous individuals under the assumption of perfect information and rational choice; nevertheless, they made quite different predictions about who forms a coalition with whom. This observation calls for careful examination of a series of simplest models that are designed to enable a focused comparison. The present study aims to do this, with particular interest in the nature of the benefit gained from the game and how it may affect the structure of coalition and the direction of natural selection. Identifying key socioecological factors should also facilitate the empirical evaluation of model predictions.

The primary objective of the present study is to provide a simple mathematical model of animal coalition that is not cognitively too demanding, but able to account for the observed diversity of coalition formation in male primates. In addition, the model is used to examine the theoretical plausibility of the social selection hypothesis in the human lineage. To these ends, this article develops three models of the three-player coalition game. All of them consider an individual’s decision on whether or not to intervene in an ongoing conflict between two individuals by providing a coalitionary aid to either one of them. The three models capture different natures of the benefit that is competed for (table 2): Model 1 supposes competition over a non-constant-sum benefit, such as dominance status, where each member of a successful coalition is expected to improve its future dominance to the same extent as a single winner; Model 2 considers a constant-sum benefit, such as reproductive opportunities, where complete scramble competition is assumed within a coalition, as a result of which the benefit is equally divided between the allies; and Model 3 deals with the case that the constant-sum benefit is divided disproportionately within a winning coalition according to their RHPs through at least in part contest-based competition. The following analysis shows that conservative and bridging coalitions are possible in all models, while revolutionary coalition is predicted only in Model 3. It is also shown that when revolutionary coalition is expected, there exist circumstances in which the intermediate and/or the weakest individuals surpass the strongest in the expected payoff, and such a reversal is enhanced by a higher synergistic effect of coalition formation and lower cost-to-benefit ratio associated with a fight.

**Table 2.** Summary descriptions of the models.

	type of benefit	division of benefit within coalitions	possible example
Model 1	non-constant sum	full benefit to each member	conflict over future dominance status
Model 2	constant sum	equal division	conflict over reproductive opportunities, followed by scramble-based competition within coalitions
Model 3	constant sum	RHP-dependent division	conflict over reproductive opportunities, followed by contest-based competition within coalitions

## 2. Models

### (a) Model 1. Non-constant-sum benefit

We consider a coalition game among three individuals, X, Y and Z, characterized by RHPs x, y and z, respectively, where X is assumed to be the strongest, followed by Y and then Z (x>y>z>0). The game is about a conflict between a pair of individuals chosen at random from the triad, where the third individual decides whether to provide coalitionary support to one individual, the other individual or none of them.

Without help from a third individual, the probability with which an individual wins a conflict against another is assumed to be proportional to its RHP. Thus, a dyadic conflict between individuals F and G, whose RHPs are f and g, respectively, is won by F with the probability given by:


(2.1) $$w_{F|G}=\frac{f}{f+g}.$$

When a third individual supports one of the conflicting individuals, the winning probability of the coalition is proportional to its RHP, given as the sum of the allies’ RHPs multiplied by s, the synergy coefficient (s>0). Thus, with the help of individual H, who has RHP h, the coalition of F and H wins a conflict against individual G with the probability given by:


(2.2) $$w_{FH|G}=\frac{s\left(f+h\right)}{s\left(f+h\right)+g}.$$

While the model allows s to take any positive value, an important difference between the cases s≥1 and 0<s<1 should be noted. When s≥1, a coalitionary support always improves the winning probability of the one being supported. On the other hand, the case 0<s<1 is paradoxical because in this case an individual’s prospect of winning may actually decline by receiving someone’s ‘help’; for example, help from individual H makes individual F less likely to win if s<f/(f+h).

Individuals gain benefit b from winning a conflict whether they win singly or jointly in a coalition (b>0); in other words, the total amount of benefit that a winning party gains is twice as large when the party is a coalition of two individuals than when it is a single individual. The non-constant-sum assumption approximates competition for dominance, where individuals are expected to improve their future dominance by winning a game to the same extent whether they win by their own or through coalitions. Another example to which the same assumption may be applied is competition for an amount of food that is larger than two individuals can consume. On the other hand, losing a conflict imposes a cost on individuals. A losing individual and each member of a losing coalition are assumed to incur the same amount of cost, c (c>0). Suppose that individuals F and G are in conflict, and individual H decides whether and to whom to provide help. Following the above assumptions, the payoffs to H when it supports F, G or nobody, are given by:


(2.3*a*) $$p_{H|F}=bw_{HF|G}-c\left(1-w_{HF|G}\right),\;\;\;\;$$

(2.3*b*) $$p_{H|G}=bw_{HG|F}-c\left(1-w_{HG|F}\right),\;\;\;\;$$

(2.3*c*) $$p_{H|\emptyset }=0,\;\;\;\;$$

respectively, where ∅ indicates that the potential helper decides not to help anyone.

Table 3 shows the payoffs to the potential helper in a game played by X, Y and Z. Three cases are distinguished according to which pair is chosen to be the conflicting individuals. First, suppose that Y and Z are in conflict. We assume that X, the potential helper, makes a decision among three possibilities (to help Y, Z or none) so as to maximize its own payoff, based on full knowledge of the RHPs of the three individuals. From table 3, we find that $p_{X|Y}>p_{X|Z}$ and $p_{X|Y}>p_{X|\emptyset }$ are satisfied if and only if:

**Table 3.** Payoffs to potential helper conditional on the choice of the recipient of the help when non-constant-sum benefit is assumed (Model 1). If a coalition wins a fight, each coalition member gains benefit b, while if it loses, each pays cost c. Potential helper *H* gains payoff $p_{H|F}$ by providing coalitionary support to *F*, where ∅ indicates that *H* supports nobody, in which case the payoff is zero.

potential helper	possible choices	payoffs
*X*	Y/Z/∅	$\begin{array}{ccc} p_{X|Y}=\frac{sb\left(x+y\right)-cz}{s\left(x+y\right)+z}, & p_{X|Z}=\frac{sb\left(x+z\right)-cy}{s\left(x+z\right)+y}, & p_{X|\emptyset }=0 \end{array}$
Y	X/Z/∅	$\begin{array}{ccc} p_{Y|X}=\frac{sb\left(x+y\right)-cz}{s\left(x+y\right)+z}, & p_{Y|Z}=\frac{sb\left(y+z\right)-cx}{s\left(y+z\right)+x}, & p_{Y|\emptyset }=0 \end{array}$
Z	X/Y/∅	$\begin{array}{ccc} p_{Z|X}=\frac{sb\left(x+z\right)-cy}{s\left(x+z\right)+y}, & p_{Z|Y}=\frac{sb\left(y+z\right)-cx}{s\left(y+z\right)+x}, & p_{Z|\emptyset }=0 \end{array}$


(2.4) $$s>\frac{kz}{x+y},$$

where k=c/b represents the cost-to-benefit ratio, and that $p_{X|\emptyset }>p_{X|Y}>p_{X|Z}$ is satisfied if and only if the inequality in (2.4) is reversed. Hence, X helps Y if (2.4) holds, and does not help anybody if the inequality is reversed. Second, consider the case when X and Z are in conflict. From table 3, $p_{Y|X}>p_{Y|Z}$ and $p_{Y|X}>p_{Y|\emptyset }$ hold if and only if (2.4) is met, and $p_{Y|\emptyset }>p_{Y|X}>p_{Y|Z}$ holds if and only if the inequality in (2.4) is reversed. Therefore, Y helps X if (2.4) is satisfied and does not help anyone if the inequality is reversed. Third, when X and Y are in conflict, we show from table 3 that $p_{Z|X}>p_{Z|Y}$ and $p_{Z|X}>p_{Z|\emptyset }$ hold if and only if:


(2.5) $$s>\frac{ky}{x+z},$$

while $p_{Z|\emptyset }>p_{Z|X}>p_{Z|Y}$ holds if and only if the inequality in (2.5) is reversed. Hence, Z helps X if (2.5) is met, and helps nobody if the inequality is reversed.

**Figure 1.**
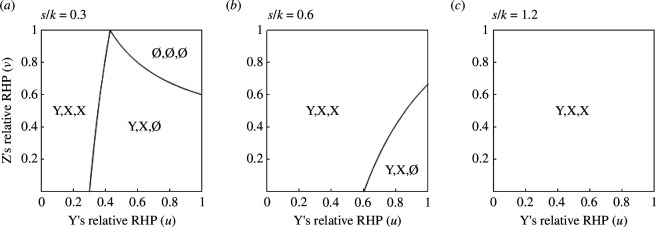
Predicted coalition structures, (∅, ∅, ∅), (Y, X, ∅) or (Y, X, X), for given combinations of Y’s and Z’s relative RHPs when (*a*) s<k/2, (*b*) k/2<s<k and (*c*) s>k (Model 1). X and Y may support mutually (bilateral conservative coalition), and Z may help X (unilateral bridging coalition) depending on parameter values. ${\overset{-}{p}}_{X}>{\overset{-}{p}}_{Y}>{\overset{-}{p}}_{Z}$ always holds so long as s≥1.

Let u and v denote the relative RHPs of Y and Z, namely, u=y/x and v=z/y, respectively (0<u<1, 0<v<1). Using (2.4) and (2.5), we specify the regions on the uv-plane that are associated with different coalition structures for a given value of s/k (for details see electronic supplementary material, Appendix A). Coalition structures are given in the form of (R_X_, R_Y_, R_Z_), designating the recipients of the coalitionary support provided by X, Y and Z, respectively. For example, (Y, X, ∅) represents the coalition structure where X helps Y, Y helps X and Z helps none. When s<k/2, three regions, each of which is associated with (∅, ∅, ∅), (Y, X, ∅) and (Y, X, X), are distinguished (figure 1*a*). When k/2<s<k, two coalition structures, (Y, X, ∅) and (Y, X, X), are possible (figure 1*b*). When s>k, (Y, X, X) is the only possibility (figure 1*c*). In sum, X and Y may help each other and Z may help X unilaterally, depending on parameter values; however, there are no combinations of parameter values for which any help is expected between Y and Z. Therefore, the model predicts conservative coalition by X and Y and bridging coalition by X and Z, but does not predict revolutionary coalition by Y and Z.

We examine the expected payoffs of the three individuals in each coalition structure. Here, we focus on the case of s≥1, where coalitionary support always improves the beneficiary’s prospect of winning. Let ${\overset{-}{p}}_{X}$, ${\overset{-}{p}}_{Y}$ and ${\overset{-}{p}}_{Z}$ denote the payoffs expected to be gained by individuals X, Y and Z, respectively, from a single game. In coalition structure (∅, ∅, ∅), we have:


(2.6*a*) $${\overset{-}{p}}_{X}=\frac{1}{3}\left(\frac{bx-cz}{x+z}+\frac{bx-cy}{x+y}\right),$$

(2.6*b*) $${\overset{-}{p}}_{Y}=\frac{1}{3}\left(\frac{by-cz}{y+z}+\frac{by-cx}{x+y}\right),\;\;\;\;$$

(2.6*c*) $${\overset{-}{p}}_{Z}=\frac{1}{3}\left(\frac{bz-cy}{y+z}+\frac{bz-cx}{x+z}\right).\;\;\;\;$$

It is straightforward to show ${\overset{-}{p}}_{X}>{\overset{-}{p}}_{Y}>{\overset{-}{p}}_{Z}$; not surprisingly, individuals with higher RHPs always enjoy higher expected payoffs in the absence of coalition formation. By similar reasoning, we also find that the same is true for coalition structures (Y, X, ∅) and (Y, X, X) so long as s≥1 (for details see electronic supplementary material, Appendix B). Therefore, the model indicates that coalition formation does not cause any reversal in the ordering of the expected payoffs among three individuals.

### (b) Model 2. Constant-sum benefit with equal division

In this subsection, we examine the case when the winning party of a conflict gains a constant sum of benefit, b, and in case the winner is a coalition of two individuals, the benefit is equally divided between them. This assumption may be relevant to male–male competition over mating opportunities, or competition for food that is rivalrous and dividable. Other assumptions are the same as in Model 1. Unequal division of benefit will be considered in Model 3. Under these assumptions, the payoffs to H when providing supports to F, G or nobody (∅), are given by:


(2.7*a*) $$p_{H|F}=\frac{b}{2}w_{HF|G}-c\left(1-w_{HF|G}\right),\;\;\;\;$$

(2.7*b*) $$p_{H|G}=\frac{b}{2}w_{HG|F}-c\left(1-w_{HG|F}\right),\;\;\;\;$$

(2.7*c*) $$p_{H|\emptyset }=0,\;\;\;\;$$

respectively.

The payoffs to a potential helper in a game played by X, Y and Z are provided in table 4. First, when Y and Z are in conflict, the payoff to the potential helper, X, has the following properties: $p_{X|Y}>p_{X|Z}$ and $p_{X|Y}>p_{X|\emptyset }$ hold if and only if:

**Table 4.** Payoffs to potential helper conditional on the choice of the recipient of the help when constant-sum benefit with equal division is assumed (Model 2). If a coalition wins a fight, constant benefit b is divided equally between the coalition members, while if it loses, each member pays cost c. Potential helper H gains payoff $p_{H|F}$ by providing coalitionary support to F, where ∅ indicates that H supports nobody, in which case the payoff is zero.

potential helper	possible choices	payoffs
X	Y/Z/∅	$\begin{array}{ccc} p_{X|Y}=\frac{\frac{sb}{2}\left(x+y\right)-cz}{s\left(x+y\right)+z}, & p_{X|Z}=\frac{\frac{sb}{2}\left(x+z\right)-cy}{s\left(x+z\right)+y}, & p_{X|\emptyset }=0 \end{array}$
Y	X/Z/∅	$\begin{array}{ccc} p_{Y|X}=\frac{\frac{sb}{2}\left(x+y\right)-cz}{s\left(x+y\right)+z}, & p_{Y|Z}=\frac{\frac{sb}{2}\left(y+z\right)-cx}{s\left(y+z\right)+x}, & p_{Y|\emptyset }=0 \end{array}$
Z	X/Y/∅	$\begin{array}{ccc} p_{Z|X}=\frac{\frac{sb}{2}\left(x+z\right)-cy}{s\left(x+z\right)+y}, & p_{Z|Y}=\frac{\frac{sb}{2}\left(y+z\right)-cx}{s\left(y+z\right)+x}, & p_{Z|\emptyset }=0 \end{array}$


(2.8) $$s>\frac{2kz}{x+y},$$

and $p_{X|\emptyset }>p_{X|Y}>p_{X|Z}$ holds if and only if the inequality in (2.8) is reversed. Thus, X supports Y if (2.8) holds, and does not support anyone if the inequality is reversed. Second, when X and Z are in conflict, we observe that $p_{Y|X}>p_{Y|Z}$ and $p_{Y|X}>p_{Y|\emptyset }$ hold if and only if (2.8) is satisfied, while $p_{Y|\emptyset }>p_{Y|X}>p_{Y|Z}$ holds if and only if the inequality in (2.8) is reversed. Therefore, Y helps X if (2.8) holds, and does not help anyone if the inequality is reversed. Third, when X and Y are in conflict, it is shown that $p_{Z|X}>p_{Z|Y}$ and $p_{Z|X}>p_{Z|\emptyset }$ if and only if:


(2.9) $$s>\frac{2ky}{x+z},$$

while $p_{Z|\emptyset }>p_{Z|X}>p_{Z|Y}$ if and only if the inequality in (2.9) is reversed. Hence, Z helps X if (2.9) holds, and does not help anyone if the inequality is reversed.

**Figure 2.**
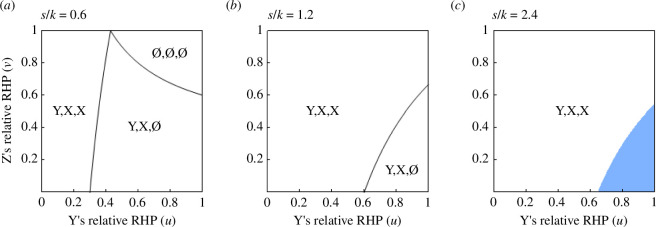
Predicted coalition structures, (∅, ∅, ∅), (Y, X, ∅) or (Y, X, X), for given combinations of Y’s and Z’s relative RHPs when (*a*) s<k, (*b*) k<s<2k and (*c*) s>2k (Model 2). As in Model 1, X and Y may support mutually (bilateral conservative coalition), and Z may help X (unilateral bridging coalition) depending on parameter values. In contrast to Model 1, ${\overset{-}{p}}_{X}>{\overset{-}{p}}_{Y}>{\overset{-}{p}}_{Z}$ may not always hold, in which case ${\overset{-}{p}}_{Y}>{\overset{-}{p}}_{X}>{\overset{-}{p}}_{Z}$ holds (the light blue region) even under s≥1. Parameter value used is s=1.0.

Using (2.8) and (2.9), we specify the regions on the uv-plane where different coalition structures are predicted for a given s/k (see electronic supplementary material, Appendix A). When s<k, three regions, each of which is associated with coalition structures (∅, ∅, ∅), (Y, X, ∅) or (Y, X, X), are distinguished (figure 2*a*). When k<s<2k, two coalition structures, (Y, X, ∅) and (Y, X, X), are possible (figure 2*b*). When s>2k, coalition structure (Y, X, X) is always predicted (figure 2*c*). These results are qualitatively the same as in Model 1, except that the threshold values of s/k are twice as large. In particular, as in Model 1, X and Y may support each other, and Z may unilaterally provide help to X, whereas in no case, coalitionary support between Y and Z is expected. That is, the model predicts conservative and bridging coalition, but not revolutionary coalition.

As for the expected payoffs of X, Y and Z when s≥1, the results are the same as Model 1 for coalition structures (∅, ∅, ∅) and (Y, X, ∅), that is, ${\overset{-}{p}}_{X}>{\overset{-}{p}}_{Y}>{\overset{-}{p}}_{Z}$ is always satisfied. In contrast, in coalition structure (Y, X, X), there exists a region on the uv-plane where ${\overset{-}{p}}_{Y}>{\overset{-}{p}}_{X}>{\overset{-}{p}}_{Z}$ holds (the light blue region in figure 2*c*) if:


(2.10) $$s<1+\frac{1}{1+2k}.$$

Hence, under the condition that (2.10) is satisfied, the model predicts a payoff reversal between X and Y, in which Y gains a higher payoff than X, for some combinations of relative RHPs (see electronic supplementary material, Appendix B).

### (c) Model 3. Constant-sum benefit with unequal division

Coalition partners may not practice equal division of the benefit. Here, we consider the case when the benefit is divided in proportion to the partners’ RHPs. This assumption seems reasonable when there is contest-based competition or bargaining within a winning coalition. Other assumptions are the same as in Model 2. Potential helper H’s payoff when it helps F, G or nobody (∅) are given, respectively, by:


(2.11*a*) $$p_{H|F}=\frac{bh}{h+f}w_{HF|G}-c\left(1-w_{HF|G}\right),\;\;\;\;$$

(2.11*b*) $$p_{H|G}=\frac{bh}{h+g}w_{HG|F}-c\left(1-w_{HG|F}\right),\;\;\;\;$$

(2.11*c*) $$p_{H|\emptyset }=0,\;\;\;\;$$

where f, g and h are the RHPs of F, G, and H, respectively.

Table 5 shows the payoffs to a potential helper in a game played by X, Y and Z. First, in a conflict between Y and Z, the following are shown. $p_{X|\emptyset }>p_{X|Y}>p_{X|Z}$ if:

**Table 5.** Payoffs to potential helper conditional on the choice of the recipient of the help when constant-sum benefit with unequal division is assumed (Model 3). If a coalition wins a fight, constant benefit b is divided between the coalition members in proportion to their RHPs, while if it loses, each member pays cost c. Potential helper H gains payoff $p_{H|F}$ by providing coalitionary support to F, where ∅ indicates that H supports nobody, in which case the payoff is zero.

potential helper	possible choices	payoffs
X	Y/Z/∅	$\begin{array}{ccc} p_{X|Y}=\frac{sbx-cz}{s\left(x+y\right)+z}, & p_{X|Z}=\frac{sbx-cy}{s\left(x+z\right)+y}, & p_{X|\emptyset }=0 \end{array}$
Y	X/Z/∅	$\begin{array}{ccc} p_{Y|X}=\frac{sby-cz}{s\left(x+y\right)+z}, & p_{Y|Z}=\frac{sby-cx}{s\left(y+z\right)+x}, & p_{Y|\emptyset }=0 \end{array}$
Z	X/Y/∅	$\begin{array}{ccc} p_{Z|X}=\frac{sbz-cy}{s\left(x+z\right)+y}, & p_{Z|Y}=\frac{sbz-cx}{s\left(y+z\right)+x}, & p_{Z|\emptyset }=0 \end{array}$


(2.12) $$s<\frac{kz}{x},$$

$p_{X|Y}>p_{X|Z}$ and $p_{X|Y}>p_{X|\emptyset }$ if:


(2.13) $$\frac{kz}{x}<s<1+\frac{k\left(x+y+z\right)}{x},$$

and $p_{X|Z}>p_{X|Y}>p_{X|\emptyset }$ if:


(2.14) $$s>1+\frac{k\left(x+y+z\right)}{x}.$$

Hence, X is predicted to support none, Y or Z when (2.12), (2.13) or (2.14) is satisfied, respectively. Second, when X and Z are in conflict, we show the following. $p_{Y|\emptyset }>p_{Y|X}>p_{Y|Z}$ if:


(2.15) $$s<\frac{kz}{y},$$

$p_{Y|X}>p_{Y|Z}$ and $p_{Y|X}>p_{Y|\emptyset }$ if:


(2.16) $$\frac{kz}{y}<s<1+\frac{k\left(x+y+z\right)}{y},$$

and $p_{Y|Z}>p_{Y|X}>p_{Y|\emptyset }$ if:


(2.17) $$s>1+\frac{k\left(x+y+z\right)}{y}.$$

It is predicted, therefore, that Y helps no one, X or Z, when (2.15), (2.16), or (2.17) holds, respectively. Third, when X and Y are in conflict, it is shown that $p_{Z|\emptyset }>p_{Z|X}>p_{Z|Y}$ if:


(2.18) $$s<\frac{ky}{z},$$

$p_{Z|X}>p_{Z|Y}$ and $p_{Z|X}>p_{Z|\emptyset }$ if:


(2.19) $$\frac{ky}{z}<s<1+\frac{k\left(x+y+z\right)}{z},$$

and $p_{Z|Y}>p_{Z|X}>p_{Z|\emptyset }$ if:


(2.20) $$s>1+\frac{k\left(x+y+z\right)}{z}.$$

Hence, Z helps no one, X or Y, when (2.18), (2.19), or (2.20) is satisfied, respectively.

**Figure 3.**
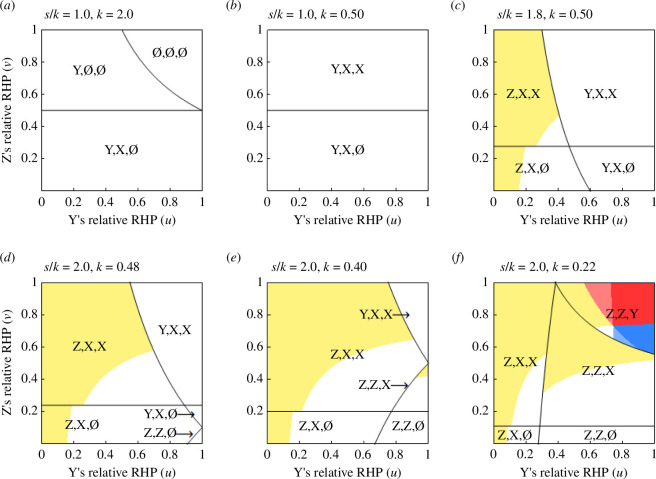
Predicted coalition structures, (∅, ∅, ∅), (Y, ∅, ∅), (Y, X, ∅), (Y, X, X), (Z, X, ∅), (Z, X, X), (Z, Z, ∅), (Z, Z, X) and (Z, Z, Y), for given combinations of Y’s and Z’s relative RHPs when (*a*) s<k, (*b*) k<s<1+k, (*c*) 1+k<s<1+2k, (*d*) $1+2k<s<s_{0}^{*}$ (see text for the definition of $s_{0}^{*}$), (*e*) $s_{0}^{*}<s<1+3k$ and s>1+3k (Model 3). Mutual support may be predicted between X and Y (bilateral conservative coalition), X and Z (bilateral bridging coalition) and Y and Z (bilateral revolutionary coalition). The ordering of the expected payoffs may be ${\overset{-}{p}}_{X}>{\overset{-}{p}}_{Y}>{\overset{-}{p}}_{Z}$ (white), ${\overset{-}{p}}_{X}>{\overset{-}{p}}_{Z}>{\overset{-}{p}}_{Y}$ (yellow), ${\overset{-}{p}}_{Y}>{\overset{-}{p}}_{X}>{\overset{-}{p}}_{Z}$ (light blue), ${\overset{-}{p}}_{Y}>{\overset{-}{p}}_{Z}>{\overset{-}{p}}_{X}$ (dark blue), ${\overset{-}{p}}_{Z}>{\overset{-}{p}}_{X}>{\overset{-}{p}}_{Y}$ (light red) or ${\overset{-}{p}}_{Z}>{\overset{-}{p}}_{Y}>{\overset{-}{p}}_{X}$ (dark red). Parameter values used are c=1.0, (*a*) s=1.0, b=0.50, (*b*) s=1.0, b=2.0, (*c*) s=1.8, b=2.0, (*d*) s=2.0, b=2.1, (*e*) s=2.0, b=2.5 and (*f*) s=2.0, b=4.6.

Using (2.12)–(2.20), we specify the conditions under which nine different coalition structures are predicted (see electronic supplementary material, Appendix A). When s<k, the uv-plane is divided into three regions, in each of which coalition structure (∅, ∅, ∅), (Y, ∅, ∅) or (Y, X, ∅) is predicted (figure 3*a*). When k<s<1+k, coalition structures (Y, X, ∅) and (Y, X, X) are possible (figure 3*b*). When 1+k<s<1+2k, the possible coalition structures are (Y, X, ∅), (Y, X, X), (Z, X, ∅) and (Z, X, X) (figure 3*c*). When $1+2k<s<s_{0}^{*}$, where $s_{0}^{*}=1/2+k+\sqrt{{\left(1/2+k\right)}^{2}+k^{2}}$, coalition structures (Y, X, ∅), (Y, X, X), (Z, X, ∅), (Z, X, X) and (Z, Z, ∅) are possible (figure 3*d*). When $s_{0}^{*}<s<1+3k$, we observe (Y, X, X), (Z, X, ∅), (Z, X, X), (Z, Z, ∅) and (Z, Z, X) (figure 3*e*). When s>1+3k, we have (Z, X, ∅), (Z, X, X), (Z, Z, ∅), (Z, Z, X) and (Z, Z, Y) (figure 3*f*).

To summarize, the model predicts a wider variety of coalition structures than Models 1 and 2; in particular, mutual support is possible not only for X and Y, but for all pairs of individuals. There exist combinations of RHP values for which mutual support between X and Z is predicted if:


(2.21) s>1+k.    

In addition to conservative and bridging coalition as observed in Models 1 and 2, revolutionary coalition is now possible in coalition structures (Z, Z, ∅), (Z, Z, X) and (Z, Z, Y). At least one of these coalition structures is possible if:


(2.22) s>1+2k.    

**Figure 4.**
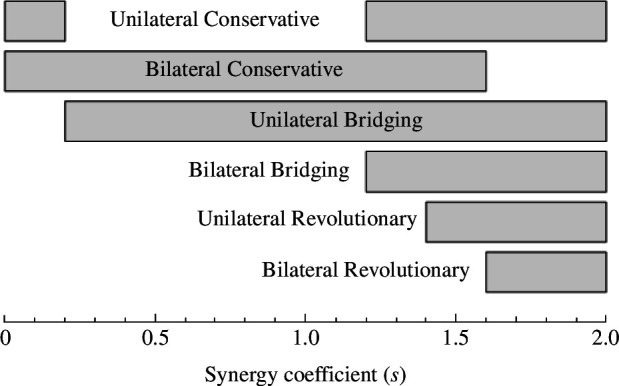
Possible types of coalition formation for different values of the synergy coefficient (s) when k=0.2 (Model 3).

Hence, (2.22) specifies a necessary condition for revolutionary coalition. Possible types of coalition formation for different values of s are shown in figure 4.

In coalition structures (∅, ∅, ∅), (Y, X, ∅) and (Y, X, X), the expected payoffs under s≥1 always satisfy ${\overset{-}{p}}_{X}>{\overset{-}{p}}_{Y}>{\overset{-}{p}}_{Z}$, which means that no payoff reversal is possible. In (Z, X, ∅), (Z, X, X), (Z, Z, ∅), (Z, Z, X) and (Z, Z, Y), a payoff reversal between Y and Z (${\overset{-}{p}}_{X}>{\overset{-}{p}}_{Z}>{\overset{-}{p}}_{Y}$) is possible for some combinations of u and v (illustrated as the yellow regions in figure 3). A payoff reversal between X and Y, causing Y to gain the highest expected payoff, is possible (${\overset{-}{p}}_{Y}>{\overset{-}{p}}_{X}>{\overset{-}{p}}_{Z}$; the light blue region) in coalition structures (Y, ∅, ∅) and (Z, Z, Y), although the condition for the payoff reversal to occur in (Y, ∅, ∅) seems to be limited. Z’s expected payoff can also exceed both X and Y (${\overset{-}{p}}_{Z}>{\overset{-}{p}}_{X}>{\overset{-}{p}}_{Y}$; the light red region) in (Z, Z, ∅), (Z, Z, X) and (Z, Z, Y). Of particular interest is whether X’s expected payoff can be the lowest among the three individuals, because if coalition formation induces selection favouring lower RHPs, that is expected to work most effectively in such a case. It turns out that this is possible in and only in (Z, Z, Y). When X is exceeded by both Y and Z in the expected payoff, the highest payoff may be achieved either by Y (${\overset{-}{p}}_{Y}>{\overset{-}{p}}_{Z}>{\overset{-}{p}}_{X}$; the dark blue region) or Z (${\overset{-}{p}}_{Z}>{\overset{-}{p}}_{Y}>{\overset{-}{p}}_{X}$; the dark red region). Coalition structure (Z, Z, Y) is predicted at least for some combinations of RHP values if:


(2.23) s>1+3k.    

Thus, (2.23) is a necessary condition for X’s expected payoff to be the lowest among the three individuals. Further details of the analysis on the expected payoffs are provided in electronic supplementary material, Appendix B.

## 3. Discussion

We have analysed three models of coalition formation, in which an individual, facing a conflict between two other individuals, decides whether and to whom to provide coalitionary support, based on the knowledge of the relative RHPs of the three individuals. One major finding is that conservative and bridging coalition can be predicted in all the models, while revolutionary coalition is possible only in Model 3 (table 6). Another finding is that in Model 1, the ordering of the expected payoff is ${\overset{-}{p}}_{X}>{\overset{-}{p}}_{Y}>{\overset{-}{p}}_{Z}$ so long as s≥1, in accordance with that of the RHP, x>y>z, while in Models 2 and 3, a reversal in the ordering of the expected payoff may occur (table 6).

**Table 6.** Summary of model predictions.

	predicted types of coalition	possible payoff reversal
Model 1: non-constant-sum benefit	bilateral conservative unilateral bridging	none
Model 2: constant-sum benefit with equal division	bilateral conservative unilateral bridging	$p_{Y}>p_{X}>p_{Z}$
Model 3: constant-sum benefit with unequal division	unilateral/bilateral conservative unilateral/bilateral bridging unilateral/bilateral revolutionary	$p_{X}>p_{Z}>p_{Y}$ $p_{Y}>p_{X}>p_{Z}$ $p_{Y}>p_{Z}>p_{X}$ $p_{Z}>p_{X}>p_{Y}$ $p_{Z}>p_{Y}>p_{X}$

The logic underlying the first finding is explained as follows. When each member of a winning coalition obtains a full amount of the benefit (Model 1) or the within-coalition division of benefit is independent of the RHP (Model 2), it is always advantageous to form a coalition with a stronger than a weaker individual. This is also true for the case of RHP-dependent division of benefit (Model 3; figure 3*a, b*), unless the synergistic effect of coalition formation (s) is large enough, or the cost-to-benefit ratio associated with a fight (k) is small enough for (2.21) to be satisfied. The predictions of Model 3 diverge from those of Models 1 and 2 when (2.21) holds true, in which case forming a coalition with a stronger individual is not necessarily advantageous anymore because a stronger ally demands a greater share of benefit. When (2.21) is satisfied, the coalition structures in which X supports Z emerge (figure 3*c–f*). When s is increased or k is decreased further, so that (2.22) is satisfied, it becomes beneficial for Y to support Z for the same reason (figure 3*d–f*). Finally, when s is so large or k is so small that (2.23) holds, the coalition structures in which Z supports Y can be predicted (figure 3*f*).

As for the second finding, there are two reasons why the stronger always gets more in Model 1. First, as already mentioned, it is always advantageous to support a stronger than a weaker individual in this model. Second, owing to the non-constant-sum assumption, the benefit does not have to be divided between allies. The situation considered in Model 2 is the same in that if an individual does help someone, it should be the stronger one. However, here the one being helped has something to lose: it has to give away half of the benefit of winning a game. As a consequence, X’s payoff may decrease when it receives unilateral help from Z to the extent that the ordering of the expected payoff between X and Y is reversed. This is most likely when Z is very weak and Y is not too weak relative to X (figure 2*c*). Underlying the apparently similar payoff reversals in Model 3 is a different mechanism. In Model 3, as s gets larger or k gets smaller, having a weak partner tends to become more advantageous, and hence a weaker individual becomes more likely to receive coalitionary support. This explains the reversal in the order of the expected payoff between Y and Z, X and Y, and X and Z (figure 3*c-f*).

The payoff reversal caused by coalition formation may drive social selection favouring reduced aggression. This possibility has been explored by Ihara [18], using the framework of a three-player coalition game as described in §1. Since Ihara [18] only considered a constant-sum benefit that is divided between coalition members in proportion to their RHPs, it should be compared with Model 3 of the present study. In contrast to Model 3, Ihara’s [18] model predicted revolutionary coalition whenever any coalition formation is included in the subgame perfect equilibrium. Since both models assume RHP-dependent within-coalition competition, forming a coalition with a stronger individual has the advantage of gaining more strength and the disadvantage of losing a greater share of benefit. It is intuitively expected, therefore, that a weaker partner should be preferred when the advantage of gaining strength relative to the disadvantage of losing benefit is larger for a weaker partner. Tan & Wang [52] formally specified, for the three-player case of their n-player coalition game, a sufficient condition for revolutionary coalition to be the unique solution. To put it in words, for a properly defined effective strength of an individual, revolutionary coalitions are predicted if the incremental gain of an individual’s effective strength from forming a coalition, divided by the partner’s effective strength, is larger for a weaker partner. If we are to apply the same logic to our case, denoting the RHPs of the focal individual and the potential partner by h and f, respectively, the relevant ratio would be $\left[s\left(f+h\right)-h\right]/f$, which decreases with increasing f. Thus, it is tempting to argue that the synergistic effect as assumed in Model 3, and Ihara [18] is inherently inclined to produce revolutionary coalitions. The reason why conservative and bridging coalitions are also predicted in Model 3 appears to be twofold. First, the present study takes the cost of losing a fight into consideration, which neither Ihara [18] nor Tan & Wang [52] did. Second, in the present study, the individual who receives help cannot choose who provides that help, whereas in Tan & Wang [52], a coalition is formed by agreement, and in Ihara [18], an individual can choose a potential helper by avoiding to attack that individual. In fact, when the cost of losing a fight approaches zero, Model 3 predicts coalition structure (Z, Z, Y) whenever s>1 (see (A34) in electronic supplementary material, Appendix B), in which a bilateral revolutionary coalition and unilateral bridging coalition are predicted, the latter of which occurs despite X not being Z’s preferred partner.

Comparison of Models 1–3 suggests that a greater diversity in the configuration of coalition is expected when the benefit of winning a conflict is divided within a coalition in a RHP-dependent manner than when it is not divided at all or divided equally. This prediction might be empirically tested if we can identify the benefits that animals gain from winning a coalitionary fight in the wild or captivity. Within Model 3, figure 4 suggests that a greater diversity in coalition configuration is expected when the synergy in coalition is more prominent for a given cost-to-benefit ratio associated with fighting. A synergy in coalition may be achieved through multiple capacities of animals, such as capacities for efficient communication, triadic awareness and sharing intentions, as well as division of labour that may result. Thus, if these capacities can be compared between species on an ordinal scale, we may be able to explore a predicted association between these capacities and the diversity in coalition configurations through species comparisons among primate males, for example. An additional interesting prediction from Model 3 that may be empirically testable is that bilateral conservative coalition and bilateral revolutionary coalition are mutually exclusive (figure 4); that is, the former configuration is possible only if the inequality in (2.23) is reversed, while the latter is possible only if (2.23) is satisfied.

So far, there has not been any attempt to test these predictions against observed data. A recent study by Neumann *et al.* [35] in wild male crested macaques (*Macaca nigra*) provides a partial opportunity to evaluate the predictions of the present study. They showed that participants of a successful coalition tended to have higher future dominance than their target. Coalitions in this species appear to be rank changing and not a mechanism to level mating distributions, suggesting the likely relevance of Model 1. Model 1 predicts conservative and bridging, but not revolutionary coalitions, which is partially supported by the observation that 88% of 128 coalitions in male crested macaques were either conservative or bridging. The infrequent occurrence of revolutionary coalitions may be explained if the positive effect of a successful coalition on the future dominance is in part RHP dependent, in which case Model 3 is to be applied. Interestingly, for participants of a revolutionary coalition, the positive effect on their future dominance was most salient when they were moderately strong in combination, while the negative effect on the target’s future dominance was most drastic when it was weakest. This pattern is expected if the positive effect on the future dominance is partially divided between coalition members in a RHP-dependent manner, so that having too strong a partner impairs the positive effect of a successful coalition.

The present study proposes socioecological circumstances in which social selection, induced by coalition formation, for reduced aggression and lowered fighting abilities is theoretically most plausible. First, coalition formation with a substantial synergy may make a weaker individual’s expected payoff higher than that of a stronger individual in Models 2 and 3, but not in Model 1, suggesting that coalition formation can potentially induce social selection in the context of competition for a constant-sum benefit, such as for mating opportunities, but not in the context of competition for a non-constant-sum benefit, such as for future dominance. This finding suggests that the reduction of male aggression in early hominins [19,20] may have been caused by coalition formation in the context of mating competition, which in part contradicts the previous theory attributing the canine reduction in *Ardipithecus* to their supposed monogamy [23]. While there is considerable debate, some researchers have argued that the degree of body size sexual dimorphism in australopithecines was not greater than in modern humans ([53,54; but see [55,56]). However, even if this is the case, the present study suggests, it does not necessarily mean that mating competition was weak in australopithecines. Second, as the payoff reversal is expected for a wide range of parameter values in Model 3, social selection is most likely when within-coalition division of benefit is RHP dependent. In other words, for social selection to work, competition needs to be at least partially contest based, as opposed to scramble based. As for male–male competition for mates, this condition may be true for most primates in which females are gregarious, likely including early hominins. Third, in Model 3, the payoff reversal is most salient when s is large, suggesting that social selection is possible when coalition formation is synergistic in such a way that a coalition of two individuals is stronger than expected from the simple sum of their strengths. As already mentioned above, a synergy in coalition may arise when individuals are capable of efficient coordination, including division of labour, in fights against other parties, which may require novel social capabilities including shared intentionality and cooperative communication [57]. This highlights an interesting possibility that the reduced canine teeth in *Ardipithecus* might be a consequence of a synergy in coalition formation based on their ability to efficiently coordinate with their allies that emerged in the earliest phase of human evolution. If this is the case, the novel social capabilities might have played the role as the background for the later emergence of characteristically human traits, such as stone-tool manufacture and brain-size expansion. An experimental work by Morgan *et al.* [58] suggests that social transmission of Oldowan stone-tool manufacture is significantly enhanced by teaching as compared with imitation or emulation. As a collaborative act of a teacher and a learner, teaching could be partially supported by the same social capabilities as those enabling synergy in coalition. Fourth, social selection can occur when the cost of losing a fight is small relative to the benefit gained from winning a fight. The cost of losing a fight, the risk of injury in particular, is expected to decrease with the average level of aggression and fighting abilities. From an evolutionary perspective, therefore, social selection for lower aggression tends to reduce the cost of fighting, which in turn facilitates further social selection, initiating a possible positive feedback loop.

To conclude, let us discuss future directions of the research. As in previous studies, we assumed that individuals make decisions so as to maximize their expected payoff. While it is theoretically possible that such an optimal partner choice is achieved by innate predispositions tailored by natural selection, it is also likely that learning is at least partially involved [59]. An extension of the models developed in the present study to explicitly incorporate learning by reinforcement should be of interest. In addition, a growing body of evidence suggests that the choice of coalition partner depends not only on the fighting abilities of individuals [60] but also on the social bonding between them [61–65]. Learning based on fighting abilities and social bonding may have different evolutionary consequences, on which further mathematical and computational investigations are worthwhile.

## Data Availability

Supplementary material is available online [66].
